# Turnover of type I and III collagen predicts progression of idiopathic pulmonary fibrosis

**DOI:** 10.1186/s12931-021-01801-0

**Published:** 2021-07-15

**Authors:** H. Jessen, N. Hoyer, T. S. Prior, P. Frederiksen, M. A. Karsdal, D. J. Leeming, E. Bendstrup, J. M. B. Sand, S. B. Shaker

**Affiliations:** 1 Biomarkers and Research, Nordic Bioscience, Herlev, Denmark; 2 Department of Respiratory Medicine, Herlev and Gentofte University Hospital, Copenhagen, Denmark; 3 Department of Respiratory Diseases and Allergy, Aarhus University Hospital, Aarhus, Denmark

## Abstract

**Background:**

Idiopathic pulmonary fibrosis (IPF) is characterized by the accumulation of fibrillar collagens in the alveolar space resulting in reduced pulmonary function and a high mortality rate. Biomarkers measuring the turnover of type I and III collagen could provide valuable information for prognosis and treatment decisions in IPF.

**Methods:**

Serological biomarkers reflecting the formation of type III collagen (PRO-C3) and degradation of type I (C1M) and III collagen (C3M) were evaluated in a real-world cohort of 178 newly diagnosed IPF patients. Blood samples and clinical data were collected at baseline, six, and 12 months. Baseline and longitudinal biomarker levels were related to disease progression of IPF (defined as ≥ 5% decline in forced vital capacity (FVC) and/or ≥ 10% decline in diffusing capacity for carbon monoxide (DLco) and/or all-cause mortality at 12 months). Furthermore, we analysed differences in percentage change of biomarker levels from baseline between patients receiving antifibrotic treatment or not.

**Results:**

Increased baseline levels of type I and III collagen turnover biomarkers were associated with a greater risk of disease progression within 12 months compared to patients with a low baseline type I and III collagen turnover. Patients with progressive disease had higher serum levels of C1M (P = 0.038) and PRO-C3 (P = 0.0022) compared to those with stable disease over one year. There were no differences in biomarker levels between patients receiving pirfenidone, nintedanib, or no antifibrotics.

**Conclusion:**

Baseline levels of type I and III collagen turnover were associated with disease progression within 12 months in a real-world cohort of IPF patients. Longitudinal biomarker levels of type I and III collagen turnover were related to progressive disease. Moreover, antifibrotic therapy did not affect type I and III collagen turnover biomarkers in these patients. PRO-C3 and C1M may be potential biomarkers for a progressive disease behavior in IPF.

**Supplementary Information:**

The online version contains supplementary material available at 10.1186/s12931-021-01801-0.

## Background

Idiopathic pulmonary fibrosis (IPF) is characterized by its unknown aetiology and poor prognosis [1, 2]. The heterogeneous rate of disease progression complicates the prediction of disease course for individual patients [3–5]. Two approved antifibrotic drugs, nintedanib and pirfenidone, have been shown to reduce the decline in forced vital capacity (FVC) compared to placebo [6, 7]. However, the only curative treatment for IPF is lung transplantation, but identification and timely referral of eligible patients can be a clinical challenge [8]. From a management and therapeutic perspective of IPF, an unfulfilled clinical need is a requirement to distinguish patients with more stable disease from those at higher risk of disease progression. The gender, age, and physiology (GAP) index is indicated to predict mortality but was not a predictor of physiological progression [9]. Hence, biomarkers that could identify patients at high risk of disease progression or stratify patients into groups more likely to benefit from antifibrotic treatment would be valuable tools.

A pathological hallmark of IPF is an extensive amount of extracellular matrix (ECM) deposition disrupting the lung architecture, resulting in reduction in lung volume and impaired gas exchange [1]. The accumulation of ECM components is a consequence of an ongoing tissue repair response to repetitive tissue injury. Among different cell types contributing to this process, the activated fibroblast produces extensive amounts of ECM proteins, such as fibrillar collagens [10, 11]. Immune cells enhance protease activity, e.g. of matrix metalloproteinases (MMPs), which can degrade collagens, and an increased MMP activity has been associated with pulmonary fibrosis [12, 13]. Direct biochemical markers that reflect the underlying collagen turnover could provide additional information about the ongoing pathological activity as compared to measures of the total protein [14].

It has been reported that serological biomarkers reflecting type I and III collagen turnover are elevated in patients with progressive compared to stable IPF [15, 16]. These biomarkers were previously measured at several time points from baseline to six months in treatment naïve IPF patients, and their trajectories beyond six months are therefore unknown. Furthermore, type I and III collagen turnover has been associated with disease progression in patients with more advanced disease than in those usually included in clinical trials, and it is therefore unknown whether this is related to disease progression in a real-world IPF cohort of more mild disease. Another unclear question is how antifibrotic treatment may influence the turnover of type I and III collagen.

The objectives of this study were to investigate (1) type I and III collagen turnover measured at baseline as prognostic biomarkers for disease progression at 12 months, (2) longitudinal assessment of type I and III collagen turnover in stable and progressive IPF patients during a 1-year period, and (3) whether antifibrotic therapy has an impact on type I and III collagen turnover.

## Materials and methods

### Patient cohort

The Pulmonary Fibrosis Biomarker (PFBIO) cohort is an ongoing, prospective cohort recruiting incident patients with IPF from two large interstitial lung disease (ILD) centers in Denmark. The PFBIO cohort has been described in details elsewhere [17]. The present study includes patients from PFBIO with data from baseline, six and 12 month visits. An overview of the participants at each visit including the type of analyses can be found in Additional file 1: Fig. S1. Patients were enrolled if they had a diagnosis of IPF according to current international guidelines [18, 19]. Patients were included immediately after their diagnosis or within maximum two months. All patients were treatment naïve at baseline. Nintedanib and pirfenidone have been available during the entire study period and the choice of which to prescribe was made by the treating clinician based on the side effect profile discussed with the patients. Patients were grouped into treated and untreated: if they had received at least one dose of nintedanib or pirfenidone, they were in the treatment group; while patients who did not receive any antifibrotic treatment were considered untreated. Clinical measures were routinely performed as part of the patients’ clinical follow-up at baseline, six, and 12 months. All patients provided written informed consent and the cohort was approved by the Regional Ethics committee (H-16001790) and the Danish Data Protection Agency (HGH-2016–017). The PFBIO study was registered at http://clinicaltrials.gov (NCT02772549) on April 29, 2016.

### Serum sampling and quantification of type I and III collagen turnover

At baseline, six, and 12 months, serum samples were collected and specific operating procedures were used to minimize variation. Briefly, blood was collected into BD Vacutainer serum silica clot activator tubes and left undisturbed at room temperature to allow the blood to clot for 30–60 min. Serum was separated by centrifugation at 1300×*g* for 10 min at 4 °C and aliquoted before storage at − 80 °C within 2 h from sample collection. Serum samples were analysed by specific competitive enzyme-linked immunosorbent assays utilizing neoepitope specific monoclonal antibodies for MMP-2,9,13 mediated degradation of type I collagen (C1M, cat. no. 1000-01), MMP-9 mediated degradation of type III collagen (C3M, cat. no. 1200-01), and for the released N-terminal pro-peptide of type III collagen (PRO-C3, cat. no. 1700-03), as previously described (Nordic Bioscience, Herlev, Denmark) [20–22].

### Statistical analyses

Baseline characteristics were compared between groups using chi-squared test, t-test or ANOVA. Disease progression was defined as an absolute decline in the percentage of predicted FVC ≥ 5% points and/or an absolute decline in the percentage of predicted DLCO ≥ 10% points and/or all-cause mortality within 12 months.

Odds ratios for disease progression within 12 months were estimated with a logistic regression model. Progression status at 12 months was used as outcome and baseline biomarker levels, age, sex, baseline FVC and DLco as covariates. The patients were divided into tertiles of biomarker levels at baseline (lower, middle and upper tertile).

A linear mixed effects model was used to examine associations between longitudinal biomarker measurements and disease progression. The model included log transformed biomarker levels as a dependent variable and progression status, visit, interaction between progression status and visit, age, and sex as covariates, and used a compound symmetry covariance structure. Missing lung function data were not imputed. The model implicitly imputes missing biomarker data and provides valid inference assuming they are missing at random. Since the missing at random assumption may be inappropriate if the biomarker data are missing due to death, sensitivity analyses were performed by restricting the study population to survivors only. Adjusted biomarker geometric means and 95% confidence interval (CI) by progression status over 12 months and p-values of the test of no difference in means between stable and progressive patients were reported.

Furthermore, differences in percentage change from baseline to follow-up between groups receiving antifibrotics or not were analysed. A similar model from the disease progression analyses was employed and included percentage change from baseline in biomarker levels as a dependent variable and treatment status (treated or untreated), visit, interaction between treatment status and visit, age, gender and C1M, C3M or PRO-C3 baseline levels as covariates.

Statistical significance was accepted at a p-value of < 0.05. All statistical analyses were conducted with R (version 4.0.4). [23].

## Results

### Baseline characteristics

A total of 178 patients diagnosed with IPF were included in the current analyses. Baseline characteristics are summarized in Table 1 and Additional file 1: Table S1. Patients not receiving antifibrotic treatment were older and had shorter 6-min walk test (6MWT) distance at baseline, despite same level of pulmonary function impairment (Table 1). Twelve months after diagnosis, 47.2% had progressive disease (Table 1). There was no difference in the number of patients with progressive disease between the different treatment groups (Table 1). There were no differences in baseline characteristics between progressive and stable IPF patients, while the change in FVC% predicted and DLco% predicted from baseline to 12 months were significant between progressive and stable IPF patients (Additional file 1: Table S1).

### Baseline levels of type I and III collagen turnover are associated with disease progression at 12 months

**Fig. 1:**
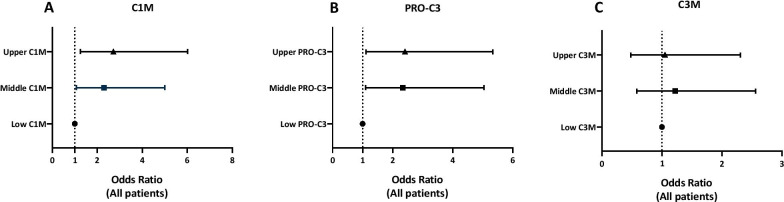
Risk of disease progression at 12 months for IPF patients. Odds ratio from IPF patients divided into tertilesfrom baseline biomarker of C1M (**A**), PRO-C3 (**B**) and C3M (**C**) are shown for the middle and upper tertilecompared to the lowest tertile. Disease progression was defined as ≥ 5% decline in FVC and/or ≥ 10% decline in DLco or all-cause mortality at 12 months. Data are presented as mean and 95% CI (error bars) adjusted for age, sex, and baseline levels of FVC and DLco. Each tertilehad n = 59–60

Analyses of patients divided into tertiles showed that compared to patients in the lower tertile, patients in the middle and upper baseline C1M tertiles had significantly higher risk of disease progression with odds ratios of 2.3 (95% CI 1.07–5.0; P = 0.033) and 2.7 (95% CI 1.26–6.0; P = 0.011), respectively (Fig. 1A). Furthermore, patients in the middle and upper baseline PRO-C3 tertiles had significantly higher risk of disease progression with odds ratios of 2.33 (95% CI 1.10- 5.05; P = 0.028) and 2.41 (95% CI 1.11- 5.33; P = 0.027), respectively, compared to patients in the lower tertile (Fig. 1B). Baseline serum levels of C3M were not related to risk of disease progression (Fig. 1C).

In subgroup analyses, adjusting for differences in treatment between the biomarker tertiles, patients in the middle and upper baseline C1M tertiles had significantly higher risk of disease progression with odds ratios of 2.29 (95% CI 1.07–5.03; P = 0.034) and 2.71 (95% CI 1.25–6.01; P = 0.012), respectively, compared to patients in the lower tertile (Additional file 1: Fig. S2A). Moreover, patients in the middle and upper baseline PRO-C3 tertiles had significantly higher risk of disease progression with odds ratios of 2.32 (95% CI 1.09–5.05; P = 0.029) and 2.33 (95% CI 1.07–5.2; P = 0.034), respectively, compared to patients in the lowest tertile (Additional file 1: Fig.S2B). Baseline serum levels of C3M were not related to risk of disease progression (Additional file 1: Fig. S2C).

### Longitudinal levels of type I and III collagen turnover products are elevated in progressive IPF

**Fig. 2:**
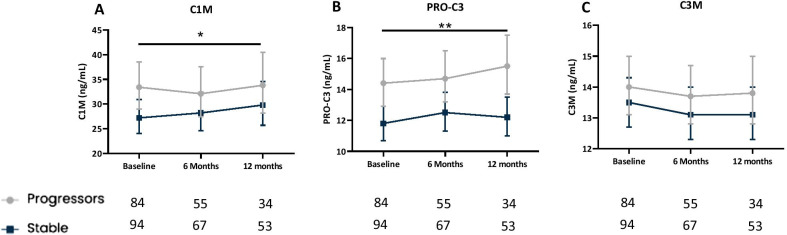
Longitudinal biomarker levels are elevated in progressive IPF patients.Serum levels of C1M (**A**), PRO-C3 (**B**) and C3M (**C**) are shown at baseline, six months and 12 months for stable (dark blue) and progressive (grey) patients with IPF. Disease progression was defined as ≥ 5% decline in FVC and/or ≥ 10% decline in DLco or all-cause mortality within 12 months. Data are presented as mean and 95% CI (error bars) adjusted for age and sex. The number of evaluable samples available for analysis at each time point is provided in the graph. The P-values for the interaction between visit and progression status for C1M (P = 0.63), PRO-C3 (P = 0.46) and for C3M (P = 0.86). Significant differences between progressive and stable patients over one year are shown as ** (P < 0.01) and * (P < 0.05)

Patients with progressive disease had significantly higher (P = 0.038) serum levels of C1M over 12 months with an average difference across all timepoints of 18% (95% CI 1–39) compared to patients with stable disease (Fig. 2A). Patients with progressive disease had significantly higher (P = 0.0022) levels of PRO-C3 over 12 months with an average difference across all timepoints of 22% (95% CI 7–38) compared to patients with stable disease (Fig. 2B). There was no significant difference for C3M between progressive and stable IPF patients (Fig. 2C). Sensitivity analyses excluding patients who died showed that patients with progressive disease had significantly higher (P = 0.029) serum levels of C1M compared to those with stable disease over 12 months with an average difference across all timepoints of 20% (95% CI 2–41) (Additional file 1: Fig. S3A). Furthermore, patients with progressive disease had significantly higher (P = 0.0055) serum levels of PRO-C3 compared to those with stable disease over 12 months with an average difference across all timepoints of 20% (95% CI 6–38) (Additional file 1: Fig. S3B). There was no significant difference for C3M between progressive and stable IPF patients (Additional file 1: Fig. S3C). Moreover, we conducted subgroup analyses in which patients were divided into groups with stable, marginal, or a significant decline in FVC. For C1M there was a significant interaction between groups and visits (P = 0.00049). The interaction is driven by an initial high average level of C1M in the marginal decline group at baseline, where the average level in the marginal decline dominates the average levels in the significant decline and stable, but at six and 12 months visits the average level in significant decline and stable dominates the average level in marginal decline (Additional file 1: Fig. S4A). There was no statistical significant difference between patients with the stable, marginal decline or significant decline in PRO-C3 or C3M levels over 12 months (Additional file 1: Fig. S4B, C).

### Nintedanib and pirfenidone did not modulate type I and III collagen turnover in IPF patients

**Fig. 3:**
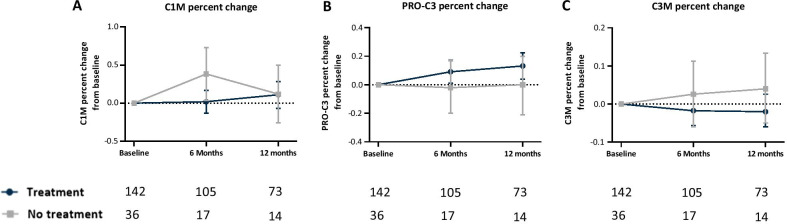
Change from baseline over time in type I and III collagen turnover in treated and untreated IPF patients. Percent change from baseline in C1M (**A**), PRO-C3 (**B**) and, C3M (**C**) at six months and 12 months for treated (nintedanib/pirfenidone) (Dark blue) and non-treated (grey) patients with IPF. Data are presented as mean and 95% CI (error bars) adjusted for age, sex and baseline levels of C1M, PRO-C3 or C3M. The number of evaluable samples available for analysis at each time point is provided in the graph. The P-values for the interaction between visit and treatment status for C1M (P = 0.18), PRO-C3 (P = 0.82) and for C3M (P = 0.79)

The change from baseline of C1M, PRO-C3 or C3M were not altered with antifibrotic treatment over 12 months compared to untreated patients (Fig. 3A–C). Furthermore, in subgroup analyses comparing patients treated with nintedanib, pirfenidone or no treatment, there were no differences in change from baseline of C1M, PRO-C3 or C3M levels over 12 months (Additional file 1: Fig. S5A–C).

## Discussion

In patients with IPF, serum biomarkers of type I and III collagen turnover measured at the time of diagnosis is related to disease progression within one year. Moreover, we demonstrated that longitudinal levels of type I and III collagen turnover could distinguish patients with progressive disease in a real-world cohort; however, we did not detect any change in collagen turnover biomarkers in patients treated with antifibrotics.

Type I and III collagen are located in the interstitial matrix and are essential components of a healthy lung by providing tensile strength to the alveolar interstitium [24]. IPF results from increased levels of interstitial collagens, changing the architecture of the airspaces in the lungs [25–27], and elevated type I and III collagen turnover has been shown to be related to disease progression in IPF patients [15, 16]. Recently, we demonstrated that type III collagen degradation (C3M) and formation (PRO-C3) are associated with disease severity at baseline and high PRO-C3 levels were related to survival [28]. In the current study, we investigated whether biomarkers of type I and III collagen turnover measured at baseline could be used to predict risk of disease progression within 12 months. When we calculated odds ratios for all IPF patients, we found that patients belonging to the middle and upper tertiles of C1M or PRO-C3 had relatively higher risk of disease progression than patients in the lower baseline tertile. These findings suggest that measuring formation of type III collagen or degradation of type I collagen at baseline could identify patients at risk of progression and help clinicians in treatment choice and timing. Currently, there is no biomarker or clinical score to predict or identify disease progression in IPF patients treated with antifibrotic therapy. Our findings suggest that future studies of type I and III collagen turnover as prognostic biomarkers in IPF are warranted. A previous study examined whether certain biomarkers (CCL13, CCL18, CXCL13, COMP, periostin, and YKL40) measured at baseline in the CAPACITY and ASCEND trials could predict FVC changes in patients receiving pirfenidone [29]. There was no association between baseline biomarker levels and change in FVC among patients treated with pirfenidone. A common limitation in biomarker studies is the lack of comparison of the prognostic performance of different biomarkers in the same population. Since our data suggest that patients with a higher baseline type I and III collagen turnover have a higher risk of disease progression, it would be interesting to make a direct comparison of C1M and PRO-C3 with the above-mentioned biomarkers to verify if they could perform better.

Interestingly, we have recently shown that degradation of type III collagen (C3M) was related to disease severity at the time of diagnosis [28]. However, in the present analyses, it was not related to increased risk of progression, indicating that C3M is more associated with disease severity rather than being a prognostic biomarker in this population. However, previous studies indicate that C3M may be a prognostic biomarker in patients with more progressive disease [15].

Next, we investigated longitudinal biomarker levels in stable and progressive IPF patients and showed that degradation of type I collagen and formation of type III collagen was elevated in patients with progressive IPF compared to stable IPF over a one year period. This is in line with results from a previous study where C1M and PRO-C3 levels were elevated at several time points from baseline to six months [15, 16]. In the present study, elevated levels of C1M and PRO-C3 were also measured at 12 months and this confirms and expands on previous findings that degradation of type I collagen and formation of type III collagen are associated with disease progression in a real-world cohort of patient with IPF and moderate disease severity. The present analyses used a less strict definition of progression to include patients with marginal physiological progression of disease, which have been shown to predict mortality in IPF patients [30–32]. Moreover, we did not find any difference in longitudinal levels of type III collagen degradation (C3M) between progressive and stable IPF patients, also in line with previous findings [16]. In contrast, it has been shown that C3M levels were increased in progressive patients [15]. The differences between the studies could be due to differences in baseline demographics among the patients recruited to the cohorts. In addition, patients in the British cohort did not receive antifibrotic treatment, while most patients in the PFBIO cohort were treated with pirfenidone or nintedanib after the baseline visit. We find it interesting that a degradation fragment of type I collagen was related to disease progression while a degradation fragment of type III collagen was not. This may be explained by the fact that C1M is generated with different matrix metalloproteinases (MMP-2, -9 and -13) and C3M is only generated with MMP-9, what may clarify the reason why C1M is related to progression and C3M is not. Degradation of type I collagen (C1M) and formation of type III and VI collagen (PRO-C3 and PRO-C6) have consistently been shown to be promising biomarkers for disease progression and these biomarkers may reflect and predict fibrotic changes in the lung [15, 16, 28]. We hope future studies will validate our findings.

Finally, we compared percentage change from baseline of type I and III collagen turnover in patients receiving antifibrotic therapy or not. Surprisingly, we did not find any differences in biomarker levels at any time points. Furthermore, we did not find any differences in biomarker levels between patients treated with nintedanib or pirfenidone. This could indicate that type I and III collagen turnover is not influenced by current antifibrotic treatments, yet the results need to be confirmed in other studies with a broader group of untreated patients. Patients without antifibrotic therapy in the present study were a highly selected group with higher age and shorter 6MWT distance compared to patients on antifibrotic therapy and this could potentially influence the results. However, our results are in line with the INMARK study where C1M and C3M were not modulated by nintedanib [33]. The patients included in the INMARK study and the PFBIO cohort had mild disease, however, the placebo group in the INMARK study showed a decline in FVC, while most patients from the PFBIO cohort had stable FVC over 12 months. In contrast to pirfenidone and nintedanib, it was shown in a small proof of concept study that treatment with omipalisib in IPF patients reduced the levels of type III collagen formation [34]. This potential antifibrotic treatment has a different mode of action in comparison to nintedanib and pirfenidone, and it is tempting to consider combinations of antifibrotics targeting different pathways of the fibrotic cascade.

A clear strength of this study is the prospective, longitudinal design which makes it possible to associate biomarkers with disease progression over time. Another advantage is the large number of participants. Patients were included in PFBIO from two of Denmark's specialized ILD centers, and this makes the study representative of a broad IPF population. In addition, a significant strength is that the collection of blood samples was standardized to minimize variation between samples. On the other hand, a weakness of the current study is that analyses were only performed in a single cohort with no validation cohort. The pulmonary function was stable for over one year in treated and untreated patients, and this may explain why the biomarker was stable over 12 months. In addition, it would be interesting to follow patients for more than a year.

In conclusion, type I and III collagen turnover measured at the time of diagnosis is related to disease progression within one year in patients with IPF. Moreover, longitudinal serum levels of type I and III collagen turnover appear to distinguish IPF patients with progressive disease from patients with stable disease. This could provide valuable information for clinicians to identify patients at high risk of disease progression. In addition, treatment with the antifibrotics nintedanib and pirfenidone does not affect serum biomarkers of type I and III collagen turnover in this population.

**Table 1:** Baseline characteristics

Parameter	All patients	Nintedanib	Pirfenidone	No treatment	P-values
N (%)	178	62 (34.8%)	80 (44.9%)	36 (20.2%)	
Age, mean (SD)	73.80 (7.53)	72.16 (7.15)	73.66 (6.87)	76.92 (8.74)	0.0097
Men, n (%)	136 (76.4%)	47 (75.8%)	62 (77.5%)	27 (75.0%)	0.95
BMI, mean (SD)	27.31 (4.51)	27.62 (4.62)	27.57 (4.16)	26.16 (5.02)	0.24
FVC (L), mean(SD)	3.04 (0.86)	3.05 (0.91)	3.08 (0.83)	2.94 (0.88)	0.7
FVC (% pred), mean(SD)	89.51 (19.52)	88.48 (19.57)	90.09 (18.71)	90.00 (21.65)	0.878
DLCO (% pred), mean(SD)	52.85 (13.25)	53.81 (12.68)	52.63 (13.13)	51.58 (14.80)	0.725
Change in FVC % pred. from baseline to 12 months, mean (SD)	0.13 (10.16)	0.20 (9.92)	0.06 (10.44)	0.15 (10.41)	0.997
Change in DLCO % pred. from baseline to 12 months, mean (SD)	− 5.03 (7.98)	− 4.70 (6.83)	− 5.37 (9.10)	− 4.85 (7.34)	0.9
6MWT (meters), mean (SD)	442.61 (105.58)	475.92 (95.20)	436.09 (106.85)	390.97 (101.61)	0.0009
Smoking status, n (%)					0.039
Never	46 (25.8%)	10 (16.1%)	25 (31.2%)	11 (30.6%)	
Active	11 (6.2%)	1 (1.6%)	6 (7.5%)	4 (11.1%)	
Former	121 (68.0%)	51 (82.3%)	49 (61.2%)	21 (58.3%)	
GAP index, n (%)					0.08
I	88 (49.7%)	34 (54.8%)	36 (45.0%)	18 (51.4%)	
II	82 (46.3%)	27 (43.5%)	42 (52.5%)	13 (37.1%)	
III	7 (4.0%)	1 (1.6%)	2 (2.5%)	4 (11.4%)	
Progression at 12 months, n (%)	84 (47.2%)	27 (43.5%)	38 (47.5%)	19 (52.8%)	0.67

*SD* standard deviation, *BMI* Body Mass Index, *FVC* forced vital capacity, *DLCO* diffusion capacity for carbon monoxide, *6MWT* six-minute walk test, *GAP* index Gender-Age-Physiology index, Disease progression was defined as an absolute decline in the percentage of predicted FVC ≥ 5% points and/or an absolute decline in the percentage of predicted DLCO ≥ 10% points and/or all-cause mortality within 12 months

## Supplementary Information

**Additional file 1.** Additional figures and table.

## Data Availability

The datasets generated and/or analysed during the current study are not publicly available due to restrictions by the Danish data protection laws but are available from the corresponding author on reasonable request.

## Abbreviations

BMI: Body mass index
CI: Confidence interval
C1M: Collage type 1 degraded by matrix metalloprotease
C3M: Collagen type 3 degraded by matrix metalloprotease
DLco: Diffusion capacity for carbon monoxide
ECM: Extra cellular matrix
FVC: Forced vital capacity
GAP index: Gender, age, and physiology index
ILD: Interstitial lung disease
IPF: Idiopathic pulmonary fibrosis
MMP: Matrix metalloprotease
PFBIO: Pulmonary Fibrosis Biomarker
PRO-C3: Collagen synthesis neoepitopes for collagen-type 3
6MWT: 6-Minute walk test
