# Supplementary material for: Turnover of type I and III collagen predicts progression of idiopathic pulmonary fibrosis
Source: Respir Res. 2021 Jul 15;22:205. doi: 10.1186/s12931-021-01801-0 (PMC8281632; doi:10.1186/s12931-021-01801-0)
Supplement: Supplementary file 1 — Additional file 1. Additional figures and table. [file 12931_2021_1801_MOESM1_ESM.pdf]

Turnover of type I and III collagen predicts progression of idiopathic pulmonary fibrosis

H. Jessen[1,2], N. Hoyer[2], T. S. Prior[3], P. Frederiksen[1], M. A. Karsdal[1], D. J. Leeming[1], E. Bendstrup[3], J. M.B. Sand[1], S. B. Shaker[2]

[1]Biomarkers and Research, Nordic Bioscience, Herlev, Denmark

[2]Department of Respiratory Medicine, Herlev and Gentofte University Hospital, Copenhagen, Denmark

[3]Center for Rare Lung Diseases, Department of Respiratory Disease and Allergy, Aarhus University Hospital, Aarhus, Denmark

Additional file 1: Table S1

Parameter	All patients	Progressive	Stable	P-values
N	178	84	94	
Age, mean (SD)	73.80 (7.53)	73.31 (7.55)	74.23 (7.53)	0.415
Men, n (%)	136 (76.4%)	69 (82.1%)	67 (71.3%)	0.126
BMI, mean (SD)	27.31 (4.51)	27.19 (4.53)	27.41 (4.51)	0.744
FVC (L), mean (SD)	3.04 (0.86)	3.13 (0.88)	2.96 (0.85)	0.17
FVC (% pred), mean (SD)	89.51 (19.52)	88.74 (20.51)	90.20 (18.67)	0.448
DLCO (% pred), mean (SD)	52.85 (13.25)	52.73 (13.31)	52.96 (13.27)	0.645
Change in FVC % pred. from baseline to 12 months, mean (SD)	0.13 (10.16)	-6.98 (7.61)	5.60 (8.34)	<0.0001
Change in DLCO % pred. from baseline to 12 months, mean (SD)	-5.03 (7.98)	-11.25 (6.90)	-0.26 (4.85)	<0.0001
6MWT (meters), mean (SD)	442.61 (105.58)	441.63 (104.92)	443.50 (106.74)	0.394
Smoking status, n (%)				0.262
Never	46 (25.8%)	17 (20.2%)	29 (30.9%)	
Active	11 (6.2%)	6 (7.1%)	5 (5.3%)	
Former	121 (68.0%)	61 (72.6%)	60 (63.8%)	
GAP index, n (%)				0.117
I	88 (49.7%)	40 (47.6%)	48 (51.6%)	
II	82 (46.3%)	38 (45.2%)	44 (47.3%)	
III	7 (4.0%)	6 (7.1%)	1 (1.1%)	

Additional file 1: Table S1: Baseline characteristics

SD standard deviation, BMI Body Mass Index, FVC forced vital capacity, DLCO diffusion capacity for carbon monoxide, 6MWT six-minute walk test, GAP index Gender-Age-Physiology index, Disease progression was defined as an absolute decline in the percentage of predicted FVC ≥ 5% points and/or an absolute decline in the percentage of predicted DLCO ≥ 10% points and/or all-cause mortality within 12 months.

Additional file 1: Fig. S1

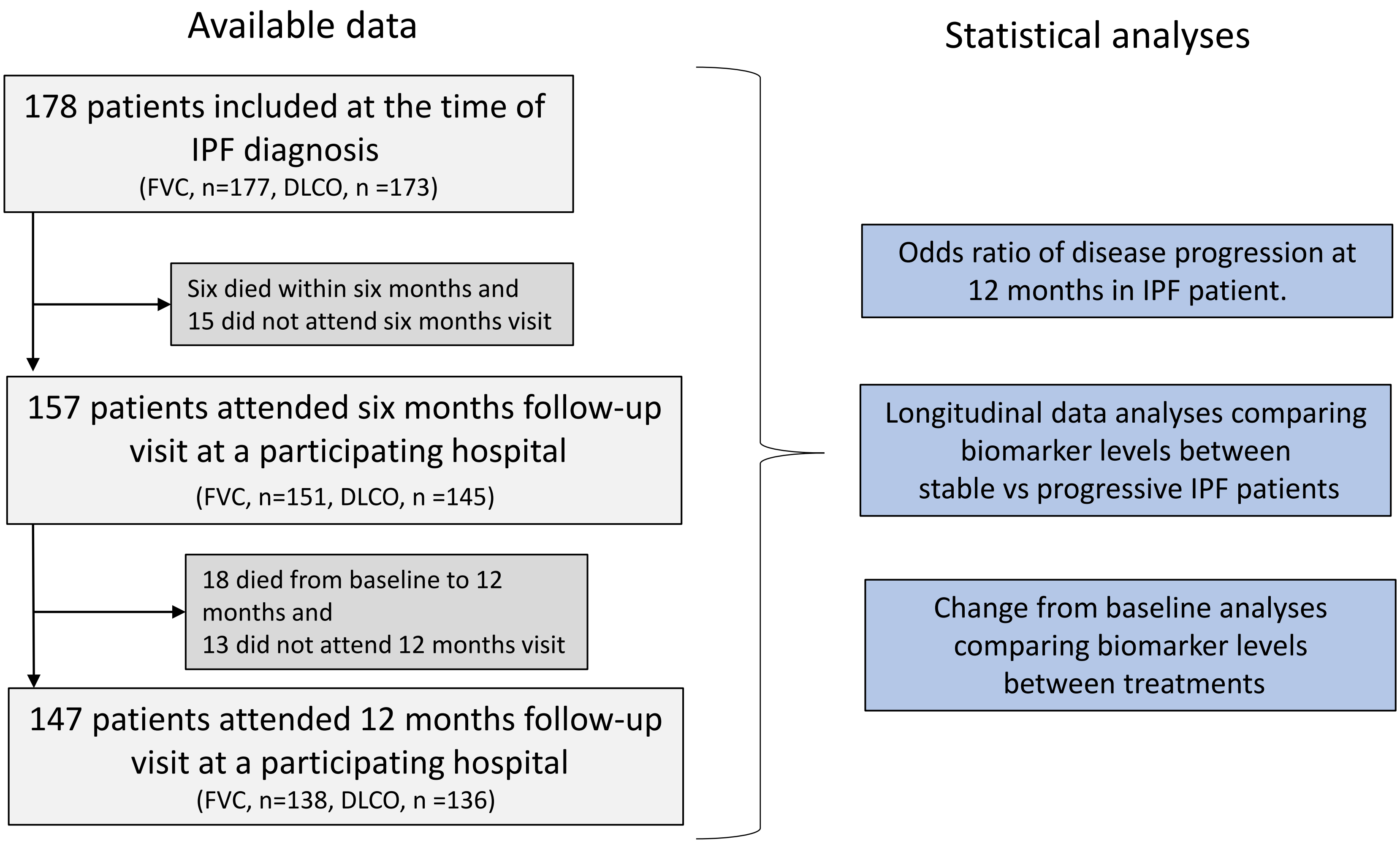

Additional file 1: Fig. S1: Flow diagram

Follow-up of patients included in the presented study.

Additional file 1: Fig. S2

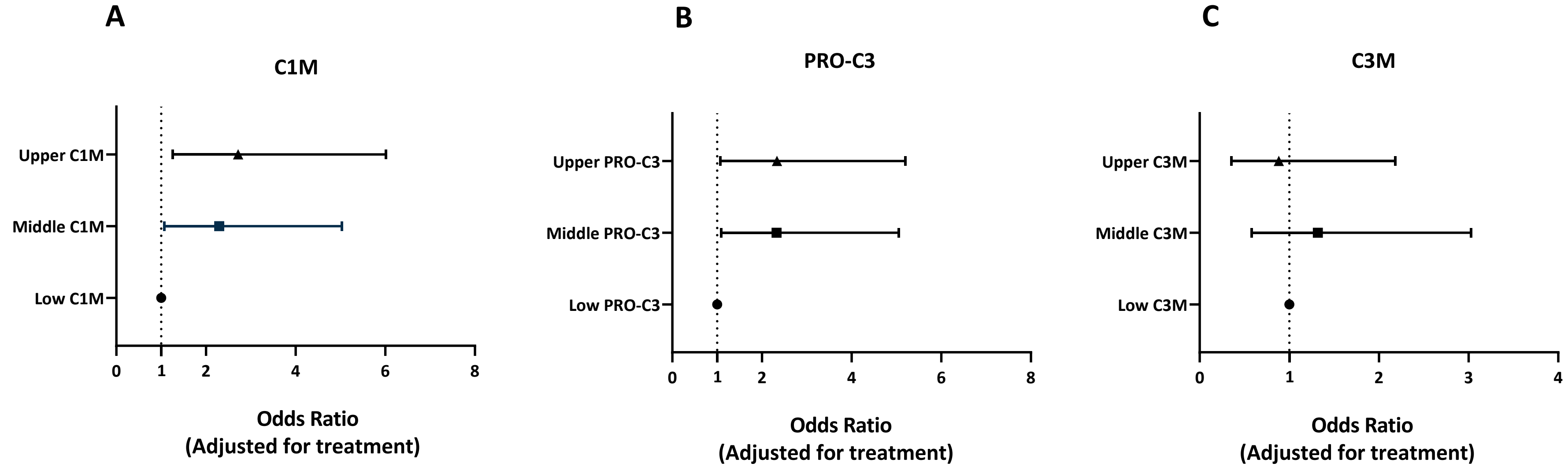

Additional file 1: Fig. S2: Risk of disease progression at 12 months for IPF patients (Adjusted for treatment).
Odds ratio from IPF patients divided into tertiles from baseline biomarker of C1M (A), PRO-C3 (B) and C3M (C) are shown for the middle and upper tertile compared to the lowest tertile. Disease progression was defined as ≥ 5% decline in FVC and/or ≥ 10% decline in DLco or all-cause mortality at 12 months. Data are presented as mean and 95% CI (error bars) adjusted for age, sex, baseline levels of FVC and Dlco, and treatment. Each tertile had n=59-60.

Additional file 1: Fig. S3

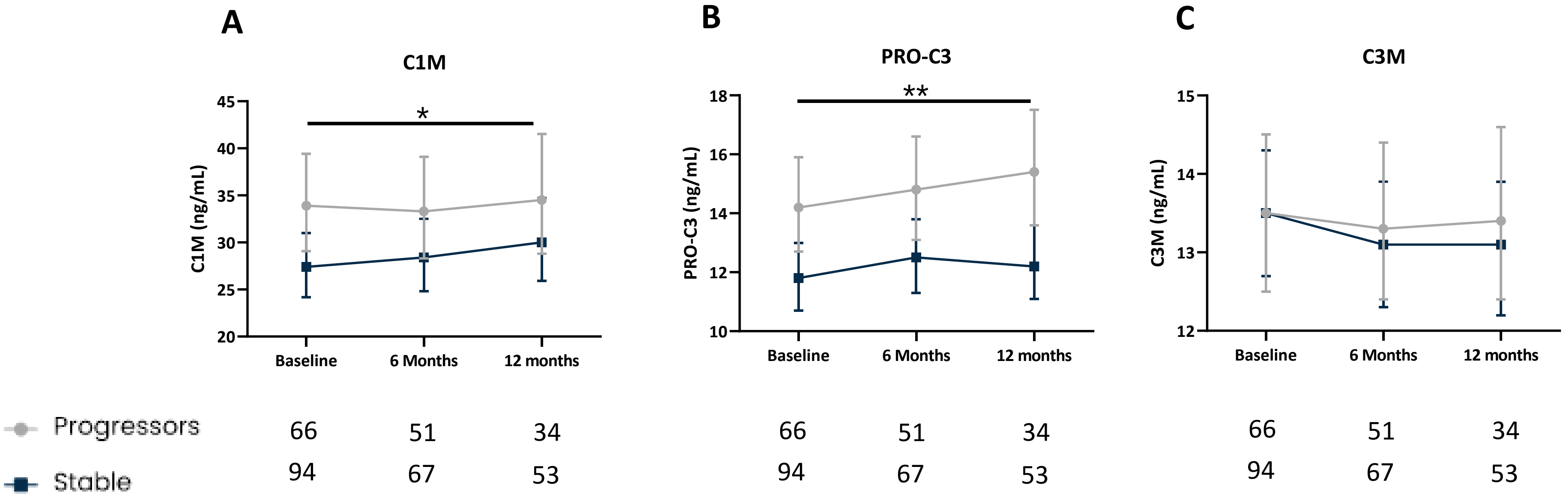

Additional file 1: Fig. S3: Longitudinal biomarker levels are elevated in progressive IPF patients (without death).
*Serum levels of C1M (A), PRO-C3 (B) and C3M (C) are shown at baseline, six months and 12 months for stable (dark blue) and progressive (grey) patients with IPF. Disease progression was defined as ≥ 5% decline in FVC and/or ≥ 10% decline in DLco within 12 months. Data are presented as mean and 95% CI (error bars) adjusted for age and sex. The number of evaluable samples available for analysis at each time point is provided in the graph. The P-values for the interaction between visit and progression status for C1M (P=0.75), PRO-C3 (P=0.52) and for C3M (P=0.73). Significant differences between progressive and stable patients over one year are shown as ** (P<0.01) and * (P<0.05).*

Additional file 1: Fig. S4

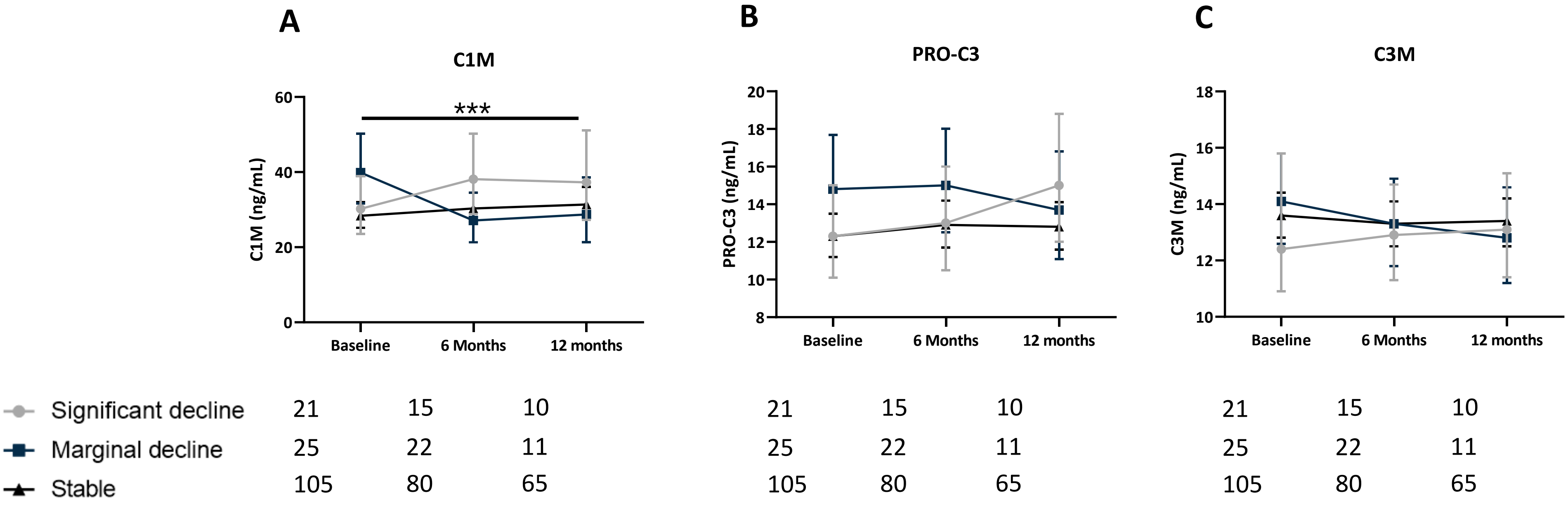

Additional file 1: Fig. S4: Longitudinal biomarker levels in stable, marginal and significant FVC decline.

*Serum levels of C1M (A), PRO-C3 (B) and C3M (C) are shown at baseline, six months and 12 months for stable (dark blue), marginal decline in FVC (black) and significant decline in FVC (grey) patients with IPF. Marginal decline in FVC was defined as ≥ 5%<10% and a significant decline ≥10% within 12 months. Data are presented as mean and 95% CI (error bars) adjusted for age and sex. The number of evaluable samples available for analysis at each time point is provided in the graph. The P-values for the interaction between groups and visits for PRO-C3 (P=0.12) and for C3M (P=0.20). Significant interaction between groups and visits are shown as *** (P<0.001).*

Additional file 1: Fig. S5

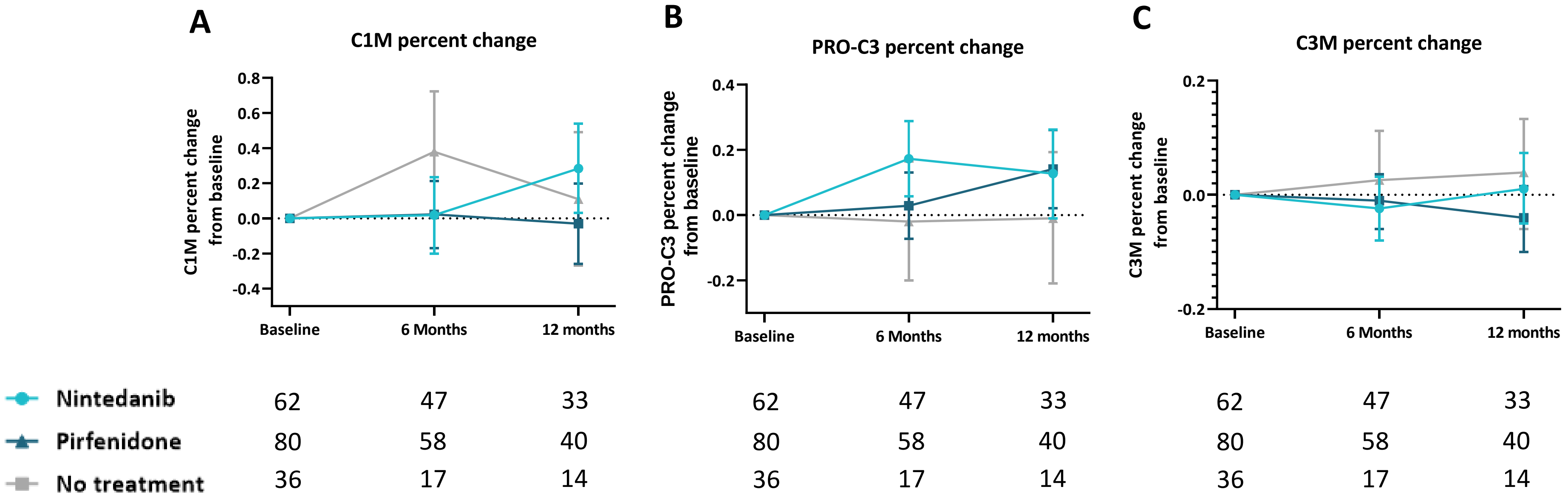

Additional file 1: Fig. S5: Change from baseline of type I and III collagen biomarkers in nintedanib, pirfenidone and untreated patients of IPF patients. *Percent change from baseline of C1M (A), PRO-C3 (B) and C3M (C) are shown at six months and 12 months for pirfenidone (dark turquoise), nintedanib (turquoise) and non-treated (grey) patients with IPF. Data are presented as mean and 95% CI (error bars) adjusted for age, sex and baseline levels of C1M, PRO-C3 or C3M. The number of evaluable samples available for analysis at each time point is provided in the graph. The P-values for the interaction between visit and treatment status for C1M (P=0.12), PRO-C3 (P=0.29) and for C3M (P=0.30).*
